# The Effect of Amniotic Membrane Transplantation on Trabeculectomy in Patients with Pseudoexfoliation Glaucoma

**DOI:** 10.1155/2022/9355206

**Published:** 2022-07-30

**Authors:** Huikyung Kim, Sangwoo Moon, Jinmi Kim, Jiwoong Lee

**Affiliations:** ^1^Department of Ophthalmology, Pusan National University College of Medicine, Busan, Republic of Korea; ^2^Department of Biostatistics, Clinical Trial Center, Biomedical Research Institute, Pusan National University Hospital, Busan, Republic of Korea; ^3^Biomedical Research Institute, Pusan National University Hospital, Busan, Republic of Korea

## Abstract

**Purpose:**

The aim is to evaluate the effect of amniotic membrane transplantation (AMT) on trabeculectomy with mitomycin C in patients with pseudoexfoliation glaucoma (PXG).

**Methods:**

This retrospective cohort study included 85 eyes of PXG who underwent trabeculectomy with or without AMT (52/33 eyes in the AMT/control group). Surgical success was defined by these criteria: (1) intraocular pressure (IOP) ≤18 mmHg and IOP reduction ≥20% and (2) IOP ≤15 mmHg and IOP reduction ≥25%. Criteria A and B defined complete success rates as patients who met these criteria without medication, respectively. Criteria C and D defined qualified success rates as patients who met these criteria with medication, respectively. Cumulative probabilities of success were compared using the Kaplan–Meier survival analysis. Cox proportional hazard models were used to evaluate the influence of AMT on surgical success accounting for confounding variables.

**Results:**

For the AMT group, compared with the control group, the complete success rates at 12 months for criterion A were 86.5% and 63.6%, respectively (*P* = 0.017) and for criterion B, 86.4% and 63.6% (*P* = 0.005). The qualified success rates at 12 months for criterion C were 92.1% and 75.1%, respectively (*P* = 0.047) and for criterion D, 92.1% and 72.1% (*P* = 0.021). On multivariable Cox regression analyses, AMT was associated with a lower failure rate on criteria A, B, and D (all *P* ≤ 0.047). Incidence of avascular bleb was higher in the control group than in the AMT group (7 vs 0 eyes; *P* = 0.004).

**Conclusions:**

In patients with PXG, trabeculectomy with AMT was associated with higher success rates and a lower incidence of avascular bleb compared with conventional trabeculectomy. *Research Registration*. This retrospective cohort study was registered at the Clinical Trial Registry of Korea (https://cris.nih.go.kr/cris/index/index.do, KCT0007228).

## 1. Introduction

Pseudoexfoliation syndrome (PXS) is characterized by abnormal production and accumulation of extracellular elastin-related microfibrillar materials in a variety of anterior eye segments including lens capsule, iris, cornea endothelium, ciliary body, and zonule [1, 2]. Pseudoexfoliation glaucoma (PXG), which develops in 30–60% of patients with PXS, is the most common cause of secondary open-angle glaucoma (SOAG) globally, accounts for 25% of patients with SOAG [1, 2].

When compared with eyes with primary open-angle glaucoma (POAG), eyes with PXG have a poorer response to medical therapy, higher mean and peak intraocular pressure (IOP), wider fluctuation of IOP, and faster glaucoma progression [2, 3]. Recent studies have reported poorer long-term outcomes in patients with PXG that underwent trabeculectomy with mitomycin C (MMC) [4, 5]. Therefore, the improvement of trabeculectomy outcomes would be considered crucial to preserving vision in patients with PXG [4–6].

When compared to POAG, eyes with PXG had a more substantial impairment of the blood-aqueous barrier after trabeculectomy [7–9], and an increased concentration of transforming growth factor-*β* (TGF-*β*) in aqueous humor [10, 11], which is known to regulate transdifferentiation of fibroblasts into myofibroblasts for wound healing and scar formation [12, 13]. Furthermore, PXG is associated with iris vasculopathy with histological degeneration of smooth muscle cells, pericytes, and endothelial cells; pseudoexfoliative material deposits at the periphery of iris vessels; micro-neovascularization, anastomotic vessels, hypoperfusion on iris angiography [14, 15]. These alterations may contribute to surgical failure after trabeculectomy in patients with PXG due to increased postoperative inflammatory reaction and overstimulated wound healing process [4, 5, 7].

The amniotic membrane has anti-fibrotic, anti-inflammatory, and anti-angiogenic properties [16–18], with the anti-scarring effect mediated by downregulating TGF-*β* signaling and suppressing myofibroblast differentiation [19, 20]. Furthermore, the amniotic membrane facilitates macrophage apoptosis and precludes polymorphonuclear cell infiltration [21].

Since Fujishima et al. reported that 13 out of 14 eyes had IOP <20 mmHg after trabeculectomy with simultaneous use of amniotic membrane transplantation (AMT) and MMC [22], several clinical investigations have reported favorable outcomes for AMT as an adjunct to trabeculectomy [23–27]. Trabeculectomy combined with AMT and MMC had a higher success rate and lower complication rate compared with conventional trabeculectomy in patients with primary or refractory glaucoma [25, 26]. In addition, the amniotic membrane placed over the scleral flap may function as a part of the bleb wall and mitigate the bleb internal pressure [28]. The development of an avascular bleb after trabeculectomy with MMC, reported as a risk factor for bleb-related infection, is associated with increased bleb internal pressure [29, 30].

Thus, we could hypothesize that concomitant use of the amniotic membrane in trabeculectomy with MMC may improve surgical outcomes and have a more desirable bleb morphology compared with conventional trabeculectomy in patients with PXG. However, no studies to date have evaluated the effect of AMT on the surgical outcome of trabeculectomy with MMC in patients with PXG and whether AMT is a prognostic factor for surgical success in these cases remains unknown. The main objective of the present study was to evaluate whether concomitant AMT affects the surgical outcomes of trabeculectomy with MMC for eyes with PXG.

## 2. Materials and Methods

This study was performed in accordance with the tenets of the Declaration of Helsinki and approved by the institutional review board of the Pusan National University Hospital (approval no. 2105-041-103). All patients gave written informed consent for the surgical procedures and for their information to be stored in the hospital database and used for research. This retrospective cohort study was conducted on 85 eyes of 85 patients with PXG who underwent trabeculectomy using MMC between August 2011 and March 2020 at the Department of Ophthalmology, Pusan National University Hospital, and was followed up for at least 12 months postoperatively. Since August 2017, we have performed the trabeculectomy with AMT for all eyes who were eligible for glaucoma surgery and agreed to receive the AMT. The indications for trabeculectomy were either increased IOP values consistently above the target on maximum tolerated medical therapy or glaucoma progression with evidence of visual field (VF) deterioration, or optic nerve change according to the treating physician.

Eyes were diagnosed as having PXG if characteristic fibrillar pseudoexfoliation materials were observed on the anterior lens surface or at the pupil margin and if they had a glaucomatous optic disc and two consecutive abnormal VF test results. Eyes that had undergone previous ocular surgery, except uncomplicated cataract extraction, with a coexisting neurological or retinal disease that could alter optic disc and affect VF, and a follow-up period <12 months were excluded.

Preoperatively, all patients underwent a complete ophthalmic examination, including measurement of the best-corrected visual acuity (BCVA), slit-lamp examination, gonioscopy, dilated funduscopy, red-free retinal nerve fiber layer and optic disc stereoscopic photographs, biometry using the IOL Master (Carl Zeiss Meditec, Dublin, CA, USA), and standard automated perimetry. The central corneal thickness (CCT) was measured using ultrasonic pachymetry (Pachmate; DGH Technology, Exton, PA), and keratometry was performed with an Auto Kerato-Refractometer (ARK-510A; NIDEK, Hiroshi, Japan). A VF test was performed using a Humphrey Field Analyzer 750i instrument (Carl Zeiss Meditec, Dublin, CA, USA) with the Swedish interactive threshold algorithm standard 24-2.

Glaucoma severity was determined by the mean deviation (MD) of the 24-2 VF. Eyes with mild glaucoma showed a VF with MD of −6 dB or more; eyes with moderate glaucoma showed a VF with MD of −6 dB to −12 dB, while eyes with severe glaucoma showed a VF with MD of less than −12 dB.

At every postoperative visit, IOP, BCVA, the number of glaucoma medications, complications, and the need for additional glaucoma surgery was evaluated. Bleb morphology at the final visit was analyzed according to the Indiana Bleb Appearance Grading Scale [31]. These standards are comprised of slit-lamp images for grading bleb height (H), extent (E), vascularity (V), and leakage graded with the Seidel test (S).

### 2.1. Surgical Technique

Under local anesthesia, the limbal conjunctiva was incised by 5-6 mm to create a fornix-based conjunctival flap, and the conjunctiva and Tenon's capsule were dissected toward the conjunctival sac. A trapezoidal scleral flap (base, 4.5 mm; apex, 2.75 mm; height, 2.75 mm) with 1/2–2/3 of the sclera thickness was then constructed. Weck-Cel sponges soaked in MMC, diluted at 0.4 mg/mL, were placed between the Tenon's capsule and sclera for 2-3 minutes, and the area exposed to MMC was irrigated with 20 mL of balanced salt solution (BSS) after removing the sponges. Anterior trabecular block resection (2 × 1.5 mm) and basal iridectomy were performed. The posterior corner of the scleral flap was secured to the sclera with two preplaced releasable sutures.

**Figure 1:**
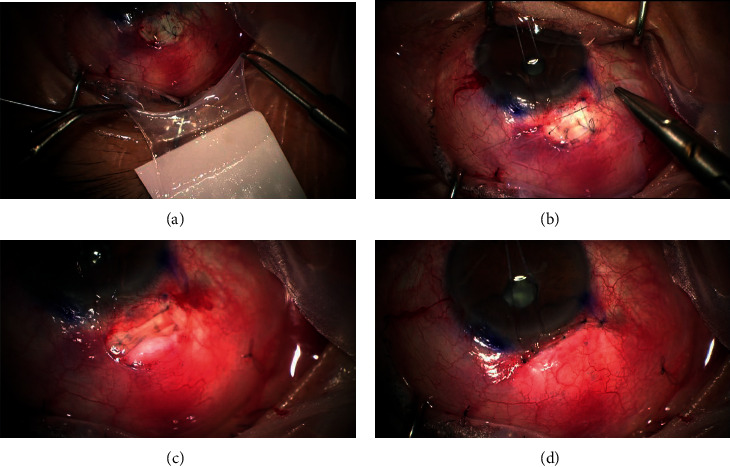
Surgical procedure of fornix-based trabeculectomy with amniotic membrane transplantation. (a) Amniotic membrane was peeled from nitrocellulose membrane. (b) Rectangular amniotic membrane was placed over scleral flap with the stromal side up. The limbal side of the amniotic membrane was secured to both sides of the scleral flap margin with two micro-point 10-0 nylon vascular needles. (c) The amniotic membrane was placed with stromal side up underneath tenon's capsule. (d) The conjunctiva and tenon's capsule were closed with interrupted micro-point 10-0 nylon vascular needles.

For trabeculectomy with AMT, a 15 × 15 mm single layer of cryopreserved amniotic membrane (MS Amnion, MS BIO inc., Seongnam, Korea) was placed on the sclera with the stromal side facing up. The limbal side of the amniotic membrane was secured to the lateral side of the scleral flap with two interrupted 10-0 nylon sutures (Ethicon Inc., Johnson & Johnson), while the fornix side of the amniotic membrane was inserted underneath Tenon's capsule and conjunctiva (Figure 1). Tenon's capsule and the conjunctiva were pulled anteriorly and closed with an interrupted suture. The anterior chamber was inflated with BSS, and the degree of aqueous outflow through the scleral flap was assessed.

Postoperatively, administration of topical eye drops, including Levofloxacin (Cravit®, Santen Pharm, Co., Osaka, Japan) four times a day and Prednisolone acetate (Predbell®, CKD Pharm, Co., Seoul, Korea) six times a day for 1 month was commenced and tapered over 8 to 12 weeks. Bleb management was performed using digital massage, releasable suture removal, or bleb needling if inadequate bleb function was noted.

### 2.2. Definition of Surgical Success

We defined surgical success using four criteria: (1) Criterion A: IOP ≤18 mmHg and IOP reduction ≥20% without medication; (2) Criterion B: IOP ≤15 mmHg and IOP reduction ≥25% without medication; (3) Criterion C: IOP ≤18 mmHg and IOP reduction ≥20% with medications; (4) Criterion D: IOP ≤15 mmHg and IOP reduction ≥25% with medications [32–34]. Surgical failure was defined as follows: (1) not meeting the above criteria on two consecutive visits; (2) the loss of light perception; and (3) the need for additional glaucoma surgery.

Hypotony was defined as IOP <5 mmHg on 2 consecutive visits at 6 weeks or later postoperatively. Prolonged hypotony was defined as IOP <5 mmHg on more than three consecutive visits and longer than 3 months [30]. Bleb leak within 1 month of surgery was defined as an early wound leak [35], and postoperative vision loss was defined as a decrease in Snellen visual acuity ≥3 lines [36].

### 2.3. Statistical Analyses

SPSS 26.0 (IBM Corp., Armonk, NY, USA) for Windows was used to perform all statistical analyses. The normality of numerical data distribution was checked with Kolmogorov–Smirnov test. Clinical characteristics between the AMT and control groups were compared using Mann–Whitney *U*-test or the Student' *t*-test test for continuous variables, and Pearson's Chi-squared test or Fisher's exact test for categorical variables. The differences between the preoperative and postoperative data were compared with Wilcoxon signed-rank test. Snellen visual acuity was converted to logarithm of the minimal angle of resolution (logMAR) equivalents for data analysis.

Kaplan–Meier survival curves were compared with the log-rank test in both groups. Cox proportional hazard models were used to estimate the influence of AMT on surgical success accounting for confounding variables. The following factors were evaluated in univariate analyses: AMT, sex, age, previous cataract surgery, preoperative number of medications, preoperative IOP, preoperative BCVA, CCT, axial length, visual field index, VF mean deviation, VF pattern standard deviation, and glaucoma stage. Variables with a *P*-value <0.20 in univariate analyses or variables clinically known to affect failure after trabeculectomy were included in multivariate analyses. A *P*-value <0.05 was considered statistically significant.

## 3. Results

The entire population consisted of 52 eyes of 52 patients (40 male and 12 female) who underwent AMT (AMT group) and 33 eyes of 33 patients (22 male and 11 female) who did not undergo AMT (control group). The mean age was 68.77 ± 8.13 years and 67.57 ± 10.29 years for the AMT and the control group, respectively. Mean follow-up time was 2.14 ± 0.83 years and 2.43 ± 0.73 years for the AMT and the control group, respectively. There were no statistically significant differences in demographics and clinical characteristics between the groups at baseline (Table 1).

**Figure 2:**
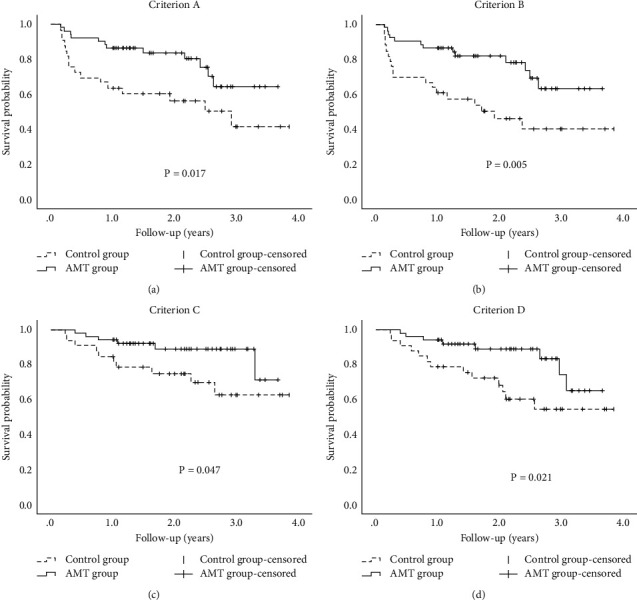
Cumulative probabilities of surgical success after trabeculectomy in the amniotic membrane transplantation (AMT) and control groups. Surgical success rates of the AMT group were significantly higher than those of the control group for all criteria (all *p*s ≤ 0.047). Criterion (a) intraocular pressure (IOP) ≤ 18mmHg and IOP reduction ≥20% without medication; criterion (b) IOP ≤15 mmHg and IOP reduction ≥25% without medication; criterion (c) IOP ≤18 mmHg and IOP reduction ≥20% with or without medications; criterion (d) IOP ≤15 mmHg and IOP reduction ≥25% with or without medications.

The cumulative success rates were 86.5% at 1 year and 83.8% at 2 years for the AMT group, and 63.6% and 56.3% for the control group by criterion A. The cumulative success rates were 86.4% at 1 year and 81.7% at 2 years for the AMT group, and 63.6% and 45.9% for the control group by criterion B. The complete success rates of the AMT group were significantly higher than those of the control group by criteria A and B (*P*=0.017, *P*=0.005, respectively) (Figure 2).

The cumulative success rates were 92.1% at 1 year and 89.0% at 2 years for the AMT group, and 75.1% and 62.8% for the control group by criterion C. Cumulative success rates were 92.1% at 1 year and 89.2% at 2 years for the AMT group, and 72.1% and 54.2% for the control group by criterion D. The qualified success rates of the AMT group were significantly higher than those of the control group by criteria C and D (*P*=0.047, *P*=0.021, respectively) (Figure 2).

Cox proportional hazard models were used to evaluate the influence of AMT on surgical failure according to each criterion accounting for confounding variables. The AMT was associated with a lower risk of surgical failure for criteria A, B, and D after adjusting for confounding variables. The adjusted hazard ratio (HR) with 95% confidence interval (CI) was 0.46 (0.21–0.99, *P*=0.047) for criterion A, HR was 0.40 (0.19–0.85, *P*=0.017) for criterion B, and HR was 0.38 (0.15–0.97, *P*=0.043) for criterion D (Table 2). The results of univariate and multivariate Cox proportional hazard models for the prediction of surgical failure are presented in the supplementary table (Available (here)).

**Figure 3:**
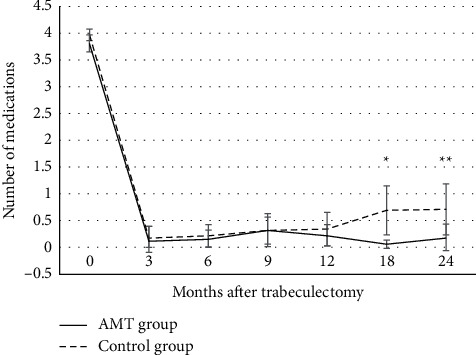
Changes in the mean number of glaucoma medications after trabeculectomy in the amniotic membrane transplantation (AMT) and control groups. The number of glaucoma medications at each time point was not significantly different between the two groups except at 18 months and 24 months after surgery. The number of glaucoma medications used after surgery was statistically lower in the AMT group than those in the control group at 18 months and 24 months postoperatively. The reduction in the number of glaucoma medications used after surgery compared with baseline was significant at all visits in both groups (all (*P*-values <0.001). The error bars are 95% confidence intervals (mean ± standard error × 1.96). ^*∗*^*P*=0.008, ^*∗∗*^*P*=0.013.

The baseline and follow-up IOP measured at 3, 6, 9, 12, 18, and 24 months after surgery was not significantly different between the two groups (*P* ≥ 0.118 for all). The number of glaucoma medications used at 3, 6, 9, and 12 months after surgery was not significantly different between the two groups (*P* ≥ 0.448 for all). The number of glaucoma medications was statistically lower in the AMT group than that in the control group at 18 and 24 months postoperatively (*P*=0.008, *P*=0.013, respectively) (Figure 3). The reduction in IOP and the number of glaucoma medications used after surgery compared with baseline was significant at all visits in both groups (*P* < 0.001 for all).

The most common complications in the AMT group were early wound leak (four eyes [7.7%]) and choroidal effusion (four eyes [7.7%]). The most common complication in the control group was hypotony (five eyes [15.2%]). All early wound leaks required conjunctival sutures except for 1 eye in the AMT group, which resolved spontaneously. Transconjunctival scleral flap suturing for hypotony was performed in 3 eyes (5.7%) in the AMT group and 2 eyes (6.0%) in the control group (*P*=1.000). Seventeen eyes (32.6%) in the AMT group and 14 eyes (26.9%) in the control group underwent bleb needling to rescue bleb function (*P*=0.364). Cataract extraction was performed postoperatively in 10 eyes (19.2%) in the AMT group and 7 eyes (21.2%) in the control group (*P*=0.824) (Table 3).

The mean BCVA (logMAR) at the final visit was 0.48 ± 0.71 and 0.53 ± 0.74 in the AMT and control groups, respectively. There were no significant changes in BCVA postoperatively in both groups (*P*=0.146, *P*=0.538, respectively). A decrease in BCVA ≥3 lines occurred in one eye (3.0%) and four eyes (7.7%) in the control and AMT groups, respectively, due to uncontrolled IOP, except for one eye in the AMT group who had no identifiable cause on examination (*P*=0.373).

Postoperative bleb morphology at the final visit is presented in Table 4. There was no significant difference in bleb height and leakage graded with the Seidel test between the two groups (*P*=0.168, *P*=0.148, respectively). The most common bleb height and leakage grades were H2 and S0 in both groups. However, the eyes of the AMT group had a broader bleb compared with those of the control group (*P*=0.019). In addition, the development of avascular bleb was observed only in the control group (seven eyes [21.2%], *P*=0.004), of which two eyes (6.1%) had multiple pinpoint leaks.

**Figure 4:**
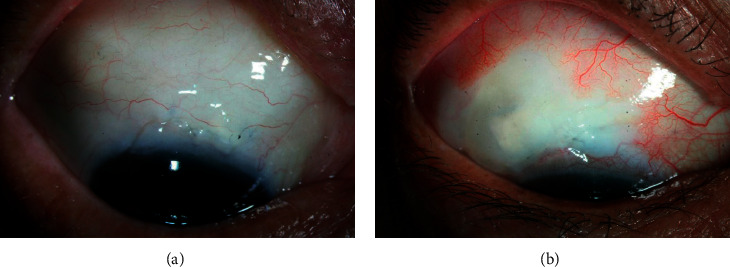
Slit-lamp photograph of the filtering bleb. (a) Bleb photograph taken 4 years after trabeculectomy with AMT showed a bleb with medium height, 4-clock hour extent, and mild vascularity. Intraocular pressure (IOP) was 13 mmHg without medication. (b) Bleb photograph taken 5 years after trabeculectomy without AMT showed medium height, 3-clock hour extent, and avascular cystic bleb. IOP was 16 mmHg without medication.

A 72-year-old male underwent a trabeculectomy with AMT on the right eye 4 years ago. The bleb photograph shows a bleb with medium height, 4-clock hour extent, and mild vascularity. IOP was 13 mmHg without medication (Figure 4(a)). A 58-year-old male underwent a trabeculectomy alone on the right eye 5 years ago. The bleb photograph shows medium height, 3-clock hour extent, and avascular cystic bleb. IOP was 16 mmHg without medication (Figure 4(b)).

## 4. Discussion

Compared with trabeculectomy with MMC alone, the concomitant use of the amniotic membrane showed a higher success rate and more desirable bleb morphology. Multivariable Cox regression analyses showed that AMT was associated with a lower failure rate after adjusting for confounding variables.

The morphologic features of the filtering blebs were different between AMT and control groups, whereby in the AMT group the blebs were broader than those in the control group, and the development of an avascular bleb was only observed in the control group. Furthermore, the control group had a progressive increase in the number of glaucoma medications used during the follow-up period, which remained constantly low in the AMT group.

The complete success rate in the control group of this study was in close agreement with those of previous studies comparing the surgical outcome of trabeculectomy with MMC between POAG and PXG [4, 5]. Li et al. reported that the success rates of trabeculectomy with MMC in PXG eyes according to the criteria of IOP <18 mmHg without medication and IOP <15 mmHg without medication at 1, 3, and 5 years to be 68%, 43%, and 29%, and 56%, 33%, and 23%, respectively; they, therefore, reported the success rate of the PXG group at 1 year after surgery to be lower than that of the POAG group with a lower target IOP [4]. Lim and Cha reported the success rates of trabeculectomy with MMC in PXG eyes according to the criteria of IOP <18 mmHg without medication, and IOP <15 mmHg without medication at 1, 3, and 5 years to be 84.4%, 39.7%, and 19.9%, and 65%, 36.9%, and 18.4%, respectively; similarly, they reported success rate in PXG patients was lower than that in POAG patients at 2 years [5].

This less favorable outcome for those with PXG may be attributable to greater damage to the blood-aqueous barrier after trabeculectomy [7]. In addition, the levels of TGF-*β*1 in aqueous humor are significantly elevated in patients with PXG compared to those with POAG or normal controls [10, 11]. TGF-*β*1 plays an essential role in postoperative scarring by triggering myofibroblast transformation through Smad/Snail pathway and stimulating the expression and synthesis of extracellular matrix components in human Tenon's capsule fibroblasts [37, 38]. Proteomic analysis of exfoliation deposits found that exfoliation materials include components that elicit complement activation, inflammatory response, and oxidative stress [39]. These findings could explain the vigorous postoperative inflammatory response and robust scar formation after trabeculectomy in eyes with PXG [4, 5, 7, 10, 11, 37–39].

However, in our study, we found that AMT-assisted trabeculectomy combined with MMC for target IOP ≤18 mmHg or ≤15 mmHg was more successful than conventional trabeculectomy with MMC alone in patients with PXG. Furthermore, we applied stricter IOP criteria to define surgical success because the results of previous studies have suggested that IOP ≤21 mmHg may be insufficient to prevent glaucoma progression in eyes with PXG [6, 40]. The multivariable Cox regression analyses confirmed that AMT was independently associated with a higher success rate.

These results are consistent with the results of earlier studies which reported that concomitant AMT under the scleral flap and/or over the sclera, with or without MMC, improved the surgical outcome of trabeculectomy [22, 23, 25–27, 41, 42]. A randomized trial found that the IOP-lowering effect of AMT-assisted trabeculectomy without MMC was comparable to trabeculectomy with MMC over 2 years and suggested the amniotic membrane as a potential alternative [43]. Other randomized prospective studies in patients with primary glaucoma or with high-risk glaucoma including neovascular, pseudophakic, and prior failure reported that trabeculectomy combined with MMC and AMT showed better surgical outcomes compared to standard trabeculectomy with MMC alone [25, 26]. Based on the results of the present and earlier studies, AMT may have a synergistic effect on the prevention of scar formation when added to trabeculectomy with MMC [26].

The amniotic membrane may serve as an anatomical barrier that prevents the postoperative adhesion of the conjunctiva and sclera because it has anti-fibrotic, anti-inflammatory, and anti-angiogenic properties [16–18]. Tseng et al. found that the levels of TGF-*β*1, *β*2, and *β*3 and TGF-*β* type II receptor transcripts, and TGF-*β*1 and *β*2 proteins in human corneal and limbal fibroblasts were suppressed in contact with an amniotic membrane stromal matrix, and down-regulation of *α*-smooth muscle actin, fibronectin, and integrin *α*5 was also observed, suggesting that an amniotic membrane stromal matrix can prevent fibroblast differentiation into myofibroblast by suppressing the TGF-*β* signaling system [19, 20]. Furthermore, the amniotic membrane may exert an anti-inflammatory effect by facilitating macrophage apoptosis and precluding polymorphonuclear cell infiltration [21]. In addition, the amniotic membrane exhibits poor immunogenicity and has a high hydraulic conductivity [16, 44].

The bleb morphology was different between the AMT and control groups in this study. The important finding of this study was that a thin avascular bleb developed in seven eyes (21.2%) of the control group and none in the AMT group. The blebs in the AMT group were significantly broader than those in the control group, which indicates a correlation between bleb function and morphology [25, 26, 42]. These findings correspond to the results of earlier studies which reported that the morphologic features of filtering blebs were different between the AMT and control groups [25, 26]. Sheha et al. found that blebs in the AMT group were diffuse, translucent with normal vascularity throughout the follow-up period, while blebs in the control group eventually became encysted avascular bleb at 9 and 12 months postoperatively [26]. Sarnicola et al. also reported that most blebs were diffuse and moderately vascularized with medium elevation after trabeculectomy with AMT [42].

The development of avascular bleb and bleb leakage after trabeculectomy with antimetabolite has been reported as a risk factor for bleb-related infection and is associated with increased internal pressure of the bleb [29, 30, 45]. The amniotic membrane placed over the scleral flap may function as a part of the bleb wall and reduce the bleb's internal pressure and the incidence of the avascular bleb, subsequently [28, 29].

There was no difference in the number of eyes that required bleb needling between the AMT and the control group in this study, which is contrary to the results from previous studies that included primary glaucoma [25, 27]. Ji et al. reported none of their 17 eyes in the AMT group required bleb needling, and Yadava et al. found that significantly more patients in their control group (7/20) required 5-FU bleb needling when compared to their AMT group (2/20) [25, 27]. However, the disease entity and number of eyes included in the previous studies were different from those of the present study, and fewer eyes with only primary glaucoma were included in their studies compared to those in our study [25, 27]. Sheha et al. found that three eyes (16.7%) with refractory glaucoma required bleb revision at 12 months after trabeculectomy with MMC [26], which could be explained by the increased wound healing in eyes with PXG compared to those with POAG [4, 5, 7, 11].

There were several limitations in our study, such as the retrospective nature of the study; however, no differences in clinical and demographic characteristics were observed between the groups. In addition, a single surgeon performed all surgeries with a standardized method. For future studies, a large prospective randomized clinical study is required to investigate the surgical outcomes of AMT-assisted trabeculectomy with MMC in patients with PXG. All patients in the study population were Asian and, therefore, the influence of AMT on trabeculectomy with MMC may be different in other populations. This study has a small sample size because it was conducted at a single center. A future study would require the control group of eyes with POAG to determine the actual beneficial effect of AMT on trabeculectomy in cases of PXG.

The clinical implication of this study in evaluating the safety and efficacy of trabeculectomy is that AMT prevents the development of a thin avascular bleb, which is a frequent complication of antimetabolite augmented trabeculectomy and a risk factor for bleb-related infection with an improvement of surgical outcomes in eyes with PXG [30]. In conclusion, the concomitant use of an amniotic membrane in trabeculectomy with MMC had a higher success rate and more desirable bleb morphology when compared to that of conventional trabeculectomy in patients with PXG.

## Figures and Tables

**Table 1:** Demographics and clinical characteristics of patients in both groups.

	AMT group	Control group	*P-*value
Number of eyes	52	33	
Follow-up time (years)	2.14 ± 0.83	2.43 ± 0.73	0.147^*∗*^
Age (years)	68.77 ± 8.13	67.57 ± 10.29	0.553^‡^
Sex			0.142^†^
Male	42 (80.8)	22 (66.7)	
Female	10 (19.2)	11 (33.3)	
Eye laterality			0.271^†^
Right	22 (42.3)	18 (54.5)	
Left	30 (57.7)	15 (45.5)	
Glaucoma stage			0.738^§^
Mild	5 (9.6)	2 (6.1)	
Moderate	7 (13.5)	6 (18.2)	
Severe	40 (76.9)	25 (75.8)	
Preoperative lens status			0.242^†^
Phakia	24 (46.2)	11 (33.3)	
Pseudophakia	28 (53.8)	22 (66.7)	
Central corneal thickness (um)	533.83 ± 32.43	543.45 ± 27.68	0.170^*∗*^
Axial length (mm)	24.20 ± 1.13	24.58 ± 1.57	0.140^‡^
Spherical equivalent (diopters)	−0.64 ± 1.82	−0.83 ± 2.58	0.910^*∗*^
Preoperative IOP (mmHg)	31.81 ± 9.17	34.15 ± 10.07	0.325^*∗*^
Number of preoperative medications	3.81 ± 0.56	3.97 ± 0.31	0.211^*∗*^
Preoperative visual acuity (logMAR)	0.46 ± 0.60	0.43 ± 0.52	0.832^*∗*^
Visual field index (%)	42.63 ± 32.09	51.52 ± 28.54	0.198^‡^
Mean deviation (dB)	−19.79 ± 8.90	−17.67 ± 7.69	0.264^‡^
Pattern standard deviation (dB)	7.68 ± 3.74	8.03 ± 3.60	0.661^‡^

Values are presented as mean ± standard deviation or number (%) AMT = amniotic membrane transplantation; IOP = intraocular pressure; logMAR = logarithm of the minimum angle of resolution. ^*∗*^ Mann–Whitney *U* test, ^‡^ Student' *t*-test, ^†^Pearson's Chi-squared test, ^§^Fisher's exact test.

**Table 2:** Crude and adjusted hazard ratios (HR) with 95% confidence intervals (CI) of amniotic membrane transplantation for surgical failure according to the four different success criteria.

	Criterion A		Criterion B		Criterion C		Criterion D	
	HR (95% CI)	*P*-value	HR (95% CI)	*P*-value	HR (95% CI)	*P*-value	HR (95% CI)	*P*-value
Model 0	0.413 (0.195–0.874)	0.021	0.376 (0.184–0.768)	0.007	0.372 (0.135–1.026)	0.056	0.369 (0.153–0.890)	0.027
Model 1	0.393 (0.184–0.837)	0.015	0.356 (0.173–0.733)	0.005	0.369 (0.132–1.032)	0.057	0.346 (0.141–0.848)	0.020
Model 2	0.421 (0.197–0.902)	0.026	0.376 (0.182–0.778)	0.008	0.406 (0.144–1.141)	0.087	0.366 (0.148–0.903)	0.029
Model 3	0.437 (0.203–0.943)	0.035	0.391 (0.189–0.811)	0.012	0.415 (0.146–1.181)	0.099	0.372 (0.147–0.942)	0.037
Model 4	0.435 (0.201–0.943)	0.035	0.396 (0.190–0.829)	0.014	0.415 (0.145–1.191)	0.102	0.353 (0.138–0.906)	0.030
Model 5	0.455 (0.209–0.990)	0.047	0.404 (0.192–0.848)	0.017	0.463 (0.158–1.351)	0.159	0.378 (0.148–0.968)	0.043

Model 0: crude; model 1: adjusted for sex; model 2: adjusted for variables in model 1 plus age; model 3: adjusted for variables in model 2 plus previous cataract surgery; model 4: adjusted for variables in model 3 plus preoperative intraocular pressure; model 5: adjusted for variables in model 4 plus glaucoma stage. The reference value of adjusted HR: sex (male).

**Table 3:** Complication after trabeculectomy in both groups.

	AMT group (*n* = 52)	Control group (*n* = 33)	*P*-value^*∗*^
Shallow anterior chamber	2 (3.8)	2 (6.1)	1.000
Early wound leak	4 (7.7)	1 (3.0)	0.644
Hyphema	2 (3.8)	1 (3.0)	1.000
Choroidal effusion	4 (7.7)	2 (6.1)	1.000
Hypotony	3 (5.8)	5 (15.2)	0.252
Decrease of visual acuity	4 (7.7)	1 (3.0)	0.644

AMT = amniotic membrane transplantation. Values are presented as number (%). ^*∗*^ Fisher's exact test.

**Table 4:** Comparison of bleb morphology based on indiana bleb appearance grading scale in both groups.

		AMT group (*n* = 52)	Control group (*n* = 33)	*P*-value^*∗*^
Bleb height (H0–H3)	0	1 (1.9)	2 (6.1)	0.168
	1	5 (9.6)	8 (24.2)	
	2	43 (82.7)	22 (66.7)	
	3	3 (5.8)	1 (3.0)	

Horizontal extent (E0-E3)	0	1 (1.9)	3 (9.1)	0.019
	1	8 (15.4)	12 (36.4)	
	2	29 (55.8)	15 (45.5)	
	3	14 (26.9)	3 (9.1)	

Vascularity (V0–V4)	0	0	1 (3.0)	0.004
	1	0	6 (18.2)	
	2	41 (78.8)	20 (60.6)	
	3	11 (21.2)	6 (18.2)	
	4	0	0	

Seidel test (S0–S2)	0	52 (100)	31 (93.9)	0.148
	1	0	2 (6.1)	
	2	0	0	

Values are presented as number (%). AMT = amniotic membrane transplantation. ^*∗*^Fisher's exact test.

## Data Availability

The data generated or analyzed during this study are available from the corresponding author upon reasonable request.

## Supplementary Materials

Supplementary Materials The results of univariate and multivariate Cox proportional hazard models for the prediction of surgical failure are presented in the supplementary table. AMT, sex, age, previous cataract surgery, preoperative number of medications, preoperative IOP, preoperative BCVA, CCT, axial length, Visual Field Index, VF mean deviation, VF pattern standard deviation, and glaucoma stage were evaluated in univariate analyses. Variables with a *P*-value <0.20 in univariate analyses were included in multivariate analyses.
