# Supplementary material for: The Effect of Amniotic Membrane Transplantation on Trabeculectomy in Patients with Pseudoexfoliation Glaucoma
Source: J Ophthalmol. 2022 Jul 30;2022:9355206. doi: 10.1155/2022/9355206 (PMC9356778; doi:10.1155/2022/9355206)
Supplement: Supplementary Materials — The results of univariate and multivariate Cox proportional hazard models for the prediction of surgical failure are presented in the supplementary table. AMT, sex, age, previous cataract surgery, preoperative number of medications, preoperative IOP, preoperative BCVA, CCT, axial length, Visual Field Index, VF mean deviation, VF pattern standard deviation, and glaucoma stage were evaluated in univariate analyses. Variables with a P-value <0.20 in univariate analyses were included in multivariate analyses. [file 9355206.f1.docx]

Supplementary Table. The results of univariate and multivariate Cox proportional hazard models for prediction of surgical failure.

|  | Criterion A | | Criterion B | | Criterion C | | Criterion D | |
| --- | --- | --- | --- | --- | --- | --- | --- | --- |
| Univariate analysis | | | | | | | | |
|  | HR (95% CI) | *P*-value | HR (95% CI) | *P*-value | HR (95% CI) | *P*-value | HR (95% CI) | *P*-value |
| AMT | 0.413 (0.195-0.874) | 0.021 | 0.376 (0.184-0.768) | 0.007 | 0.372 (0.135-1.026) | 0.056 | 0.369 (0.153-0.890) | 0.027 |
| Sex | 0.771 (0.292-2.033) | 0.599 | 0.814 (0.333-1.987) | 0.651 | 1.163 (0.374-3.616) | 0.794 | 0.879 (0.295-2.624) | 0.818 |
| Age | 0.964 (0.928-1.001) | 0.058 | 0.968 (0.934-1.004) | 0.079 | 0.958 (0.912-1.007) | 0.092 | 0.971 (0.928-1.017) | 0.213 |
| Previous cataract surgery | 1.340 (0.618-2.906) | 0.458 | 1.392 (0.666-2.909) | 0.379 | 1.230 (0.445-3.398) | 0.690 | 1.279 (0.525-3.114) | 0.588 |
| Preoperative number of medications | 1.714 (0.540-5.446) | 0.361 | 1.127 (0.483-2.626) | 0.782 | 1.273 (0.341-4.761) | 0.720 | 1.374 (0.388-4.863) | 0.623 |
| Preoperative IOP | 1.041 (0.999-1.086) | 0.055 | 1.045 (1.005-1.087) | 0.026 | 1.032 (0.980-1.087) | 0.230 | 1.028 (0.983-1.075) | 0.226 |
| Preoperative BCVA | 1.106 (0.622-1.965) | 0.731 | 1.197 (0.692-2.071) | 0.519 | 0.961 (0.395-2.338) | 0.929 | 0.938 (0.428-2.057) | 0.873 |
| CCT | 1.008 (0.995-1.020) | 0.229 | 1.005 (0.994-1.017) | 0.395 | 1.001 (0.985-1.017) | 0.887 | 1.003 (0.989-1.017) | 0.658 |
| Axial length | 0.988 (0.756-1.292) | 0.932 | 0.991 (0.760-1.291) | 0.945 | 1.065 (0.755-1.502) | 0.720 | 1.006 (0.732-1.384) | 0.970 |
| VFI | 0.999 (0.987-1.011) | 0.867 | 0.999 (0.988-1.011) | 0.912 | 0.997 (0.981-1.014) | 0.741 | 0.999 (0.985-1.013) | 0.910 |
| VF MD | 0.996 (0.954-1.041) | 0.874 | 0.998 (0.958-1.041) | 0.939 | 0.987 (0.931-1.048) | 0.678 | 0.996 (0.947-1.047) | 0.865 |
| VF PSD | 1.004 (0.908-1.110) | 0.937 | 1.011 (0.919-1.112) | 0.824 | 1.023 (0.892-1.174) | 0.745 | 1.041 (0.923-1.175) | 0.512 |
| Glaucoma stage | 1.085 (0.592-1.987) | 0.793 | 1.172 (0.640-2.147) | 0.607 | 1.538 (0.570-4.150) | 0.395 | 1.363 (0.639-2.909) | 0.424 |
|  | Criterion A | | Criterion B | | Criterion C | | Criterion D | |
| Multivariate analysis | | | | | | | | |
|  | HR (95% CI) | *P*-value | HR (95% CI) | *P*-value | HR (95% CI) | *P*-value | HR (95% CI) | *P*-value |
| AMT | 0.444 (0.207-0.949) | 0.036 | 0.409 (0.198-0.845) | 0.016 | 0.395 (0.142-1.097) | 0.075 | 0.369 (0.153-0.890) | 0.027 |
| Age | 0.960 (0.923-0.999) | 0.042 | 0.965 (0.930-1.002) | 0.062 | 0.964 (0.920-1.011) | 0.130 |  |  |
| Preoperative IOP | 1.050 (1.006-1.095) | 0.024 | 1.050 (1.009-1.092) | 0.016 |  |  |  |  |

AMT, amniotic membrane transplantation; BCVA, best-corrected visual acuity; CCT, central corneal thickness; CI, confidence interval; HR, hazard ratio; IOP, intraocular pressure; MD, mean deviation; PSD, pattern standard deviation; VF, visual field; VFI, Visual Field Index
